# Recent advances in understanding assembly of the primary cilium membrane

**DOI:** 10.12703/r/10-16

**Published:** 2021-02-22

**Authors:** Saurabh Shakya, Christopher J Westlake

**Affiliations:** 1 Center for Cancer Research, National Cancer Institute, National Institutes of Health, Frederick, Laboratory of Cellular and Developmental Signaling, Frederick, MD 21702, USA

**Keywords:** Ciliogenesis, primary cilium, Rab, membrane trafficking, ciliopathy

## Abstract

Ciliogenesis describes the assembly of cilia in interphase cells. Several hundred proteins have been linked to ciliogenesis, which proceeds through a highly coordinated multistage process at the distal end of centrioles requiring membranes. In this short review, we focus on recently reported insights into the biogenesis of the primary cilium membrane and its association with other ciliogenic processes in the intracellular ciliogenesis pathway.

## Introduction

Human cells typically have a single immotile primary cilium or one or more motile cilia. The primary cilium functions in multiple signal transduction pathways needed for normal development and tissue homeostasis^1,2^. Defects in the function of this organelle cause genetic disease, referred to as ciliopathy.

The primary cilium develops from the mother centriole (MC) and is structurally comprised of a microtubule-based axoneme surrounded by a ciliary membrane, which serves as the chief signaling hub for the organelle. A transition zone (TZ) at the cilium base acts as a molecular gate to regulate entrance and exit of proteins and lipids^3,4^. Notably, the majority of ciliopathy-linked proteins localize to the TZ^2,5^. Intraflagellar transport (IFT) proteins were the first proteins shown to function in ciliogenesis, and both IFT-A and IFT-B complexes regulate anterograde and retrograde transport along the axoneme^6–10^. Although many other proteins have been shown to be important for ciliogenesis, we still have only a very basic understanding of the mechanisms involved in this process.

## Ciliogenesis mechanisms

**Figure 1.**
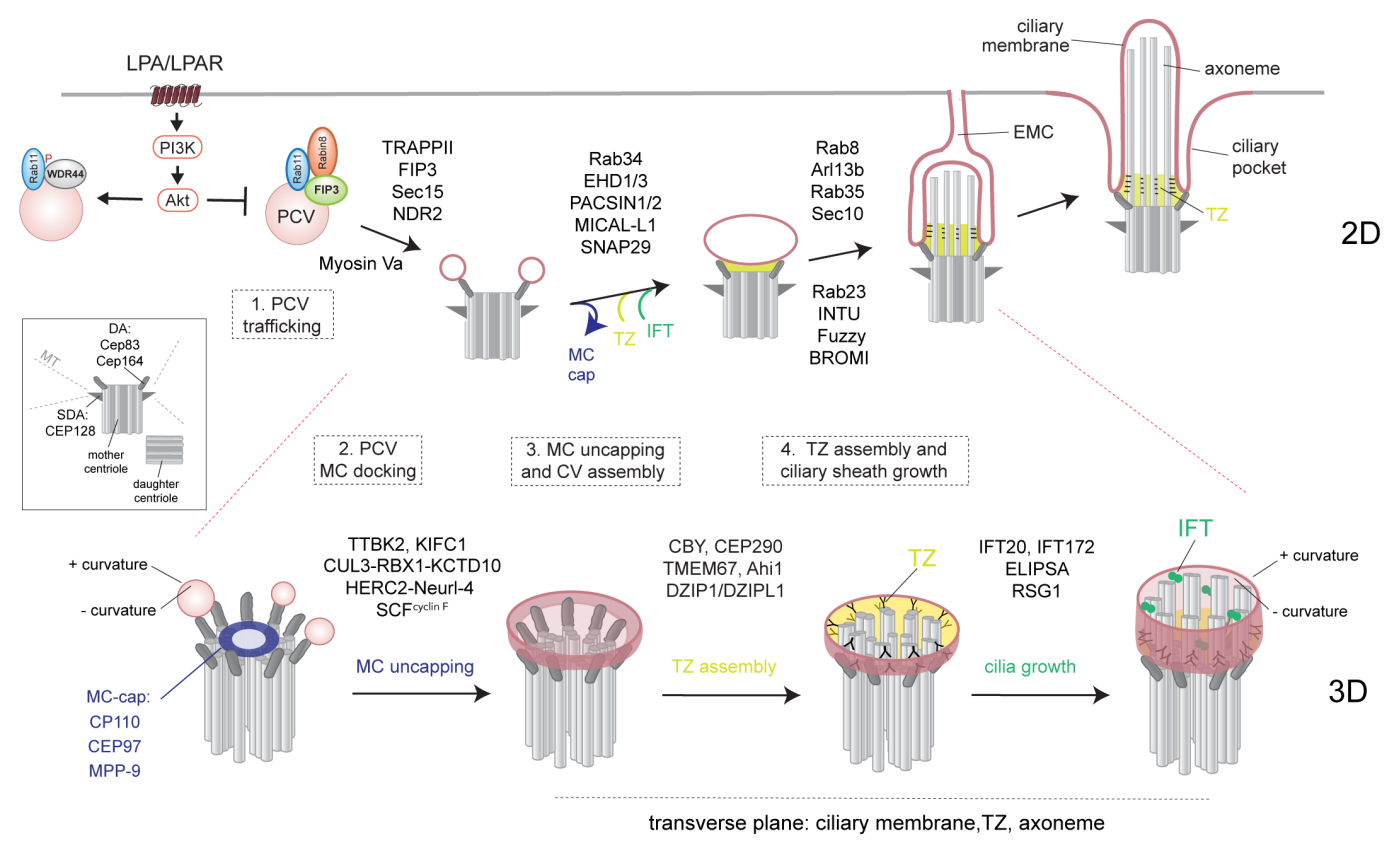
Membrane trafficking regulation of primary cilium assembly in intracellular pathway. Membrane trafficking regulation of ciliogenesis stages illustrated in two dimensions (2D) (top) and three dimensions (3D) (bottom). Ciliogenic factors linked to membrane trafficking are shown for ciliogenesis stages associated with (1) mitogen signaling regulation of preciliary vesicle (PCV) trafficking to the mother centriole (MC), (2) docking of PCVs to the MC mediated by subdistal appendage (SDA) and distal appendage (DA) proteins (inset), (3) assembly of the ciliary vesicle (CV) from PCVs and associations with the ubiquitin degradation of the MC cap, and (4) coordination of ciliary sheath membrane growth and transition zone (TZ) and axoneme assembly. The developing intracellular cilium can emerge from the cell surface via the formation of an extracellular membrane channel (EMC) which develops from the ciliary membrane to plasma membrane (gray line). Pink membranes show the developing ciliary membrane and ciliary pocket, and + and – curvature indicates membrane surface shape. IFT, intraflagellar transport; MT, microtubule.

The assembly of the cilium is thought to occur by two mechanisms: an extracellular pathway and an intracellular pathway. Polarized epithelial cells use the extracellular pathway, whereas fibroblasts and mesenchymal cells follow the intracellular scheme^11,12^. In both pathways, docking of cellular membranes to the distal appendages (DAs) on the MC (Figure 1) triggers the removal of proteins from the MC, referred to as MC uncapping, needed for axoneme assembly^13^. This centriole cap controls the elongation of these microtubule triplet-based structures^14^. In the extracellular ciliogenesis pathway, the MC docks directly to the plasma membrane (PM) through distal appendage proteins (DAPs) on the DAs^15^, although there are many outstanding questions about the initiation mechanisms involved in this process. Most recently, it was suggested that the cytokinesis midbody remnant directs MC docking to the PM^16^. In contrast, in the intracellular ciliogenesis pathway, the cilium is assembled at least partially in the cytoplasm and involves the trafficking of membrane vesicles from the endocytic recycling compartment (ERC) and the Golgi to the MC^15,17–19^ (Figure 1). Preciliary vesicles (PCVs) dock to DAPs, where they have been referred to as distal appendage vesicles (DAVs), and subsequently fuse to form a larger ciliary vesicle (CV) covering the distal end of the MC^15,18,20,21^. This process triggers MC uncapping followed by the growth of the axoneme surrounded by a double-membrane sheath that develops from the CV.

## Ciliogenesis initiation and the Rab11–Rab8 cascade

The earliest mechanistic evidence showing membrane trafficking regulator associations with the intracellular pathway came from the observation that a Rab11–Rab8 cascade functions in ciliogenesis^18,22–24^. Rabs are members of the Ras superfamily of small GTPases and are master regulators of membrane trafficking important in the biogenesis, transport, tethering, and fusion of membranous structures in the cell^25^. Rab11 organizes the trafficking of PCVs from the ERC and transports Rabin8, a Rab8 guanine nucleotide exchange factor (GEF), to the MC in order to activate Rab8 to grow the ciliary membrane at the CV stage^18,20^. This preciliary trafficking process occurs within minutes of cues for cells to ciliate, suggesting that this is a key step in ciliogenesis initiation^18^. Recently, we showed that Rabin8 PCV-dependent trafficking and ciliogenesis initiation are negatively regulated by lysophosphatidic acid (LPA)/LPA receptor 1 (LPAR1)-dependent activation of the PI3K-Akt signaling pathway in cultured cells^26^. Akt phosphorylates the Rab11 effector WDR44 and stabilizes its binding to Rab11, thus preventing the formation of an effector complex between Rab11 and FIP3 needed to traffic Rabin8-containing PCVs to the MC.

## Docking preciliary vesicles to the mother centriole

How PCVs are trafficked by the cytoskeleton to the MC DAs remains unclear, although the Golgi-associated motors dynein-2, Myosin Va, and kinesin-14 (KIFC1) have been linked to ciliogenesis^19,21,27–29^. Preciliary trafficking of Rabin8-containing PCVs requires microtubules^18^, yet microtubules are likely not anchored to DAs but are observed on more proximal subdistal appendages (SDAs) on the MC^30^ (Figure 1). This raises the possibility that PCVs are first trafficked to the SDAs and then shuttled to the DAs. Indeed, the SDA protein CEP128 has been shown to mediate Rab11 association with the MC^31^ while Rabin8 can interact with the DAP CEP164^32^. PCV transport from SDAs to DAs could be assisted by large-molecular-weight membrane trafficking regulator complexes, including TRAPPII and the exocyst, both of which are required for ciliogenesis and interact with Rabin8 through their respective components TRAPPC14 and Sec15^18,33,34^. This idea is supported by the observation that TRAPPC14 interacts with the DAPs CEP83 and FBF1^33^. The more proximal DA position of CEP83, closer to the SDA, could enable a “hand-off” of PCVs to the distally localized DAP CEP164, a presumed anchor point for the ciliary membrane^35^.

Pericentriolar PCV transport to the MC has also been recently reported to be regulated by the motor protein Myosin Va^21^. This mechanism is independent of Rabin8 preciliary trafficking, suggesting that more than one type of PCV may be involved in cilium assembly^33^. Consistent with this idea, several other Rabs have been linked to ciliogenesis (Rab10, -23, -29, -34, and -35), although their precise functions are poorly understood^24,36–41^. Interestingly, in contrast to Rab8, its closest homolog Rab10 was recently shown to block ciliogenesis through a phosphorylation-dependent process involving the shared effector RILPL1, which localizes to the MC and prevents ciliogenesis^42^. These findings differ from an earlier study showing that Rab8 and Rab10 have overlapping ciliogenesis functions^41^. Interestingly, both Rabs are activated by Rabin8^43,44^, suggesting that regulation of its GEF function could be important for Rab8 and Rab10 opposing ciliogenesis roles.

## Ciliary vesicle assembly

Following docking of PCVs to the MC, the Rab11- and Rab8-associated endosomal membrane trafficking regulators EHD1 and EHD3, along with their binding partners MICAL-L1 and the F-BAR membrane-shaping protein PACSIN1 and -2, function in the assembly of the CV^20,45–47^. F-BAR–containing proteins are involved in sensing or establishing membrane curvature or both^48^. EHD proteins may also influence membrane shape directly or indirectly by recruiting PACSINS to membranes and assist with membrane fusion^47,49^. EHD1 recruits both PACSINs and SNAP29, a SNARE membrane fusion regulator^20^, to DAVs, while Rab34 was recently reported to mediate EHD1 association with MC-associated ciliogenic membranes^36^. Together, these observations support a model wherein DAVs fuse to form the CV, a process that appears to require membrane shaping.

## Membrane trafficking regulator links to mother centriole uncapping

Ciliogenic EHD and PACSIN proteins and MICAL-L1 are required for MC uncapping, suggesting that this process is dependent on CV assembly^20,45–47^, although inexplicitly Rab34 is dispensable for MC cap removal^36^. The MC cap is comprised of three proteins: CP110, CEP97, and M-phase phosphoprotein 9 (MPP9)^14,50^. MC uncapping involves several proteasomal degradation regulators and the tau tubulin kinase 2 (TTBK2)^14,51,52^. Whereas some of these factors localize to the MC, others are recruited upon initiation of ciliogenesis, suggestive of directed transport mechanisms. TTBK2 recruitment to the MC depends on the motor KIFC1^19^ and changes in PtdIns(4)P levels^53^, a lipid enriched in the ERC and Golgi-derived vesicles^54,55^. New insights into the initiation of MC uncapping have recently emerged from characterizations of MPP9^50^. The motor KIF24 maintains MPP9 at the MC under non-ciliating conditions, and following ciliogenesis initiation, TTBK2 is recruited to the MC where it phosphorylates MPP9 at Ser^629^, resulting in its removal by an unknown mechanism. MPP9 loss from the MC cap could trigger a cascade whereby CEP97 and CP110 are degraded. The E3 ligase complex CUL3-RBX1-KCTD10 degrades CEP97^52^, and CP110 degradation has been linked to the ubiquitin ligases HERC2 and SCF^cyclinF14,56,57^. Interestingly, the HERC2 cofactor Neurl-4 translocates from the daughter centriole to MC during ciliogenesis^58^. Thus, it seems plausible that PCVs could be involved in trafficking this and other factors needed for MC uncapping.

## Transition zone and ciliary membrane assembly

TZ protein recruitment to the MC also coincides with the DAV-to-CV assembly stage, suggesting a role for PCVs in transporting TZ factors^20,45^. Interestingly, the TZ protein Chibby (CBY) stabilizes Rabin8 and CEP164 associations^59^, suggesting that this TZ protein could affect PCV docking to DAPs or regulate downstream Rab8 activation or both. Similarly, the TZ protein CEP290 is important for recruiting Rab8 to the developing cilium^60^. Importantly, mutation or ablation of CBY and CEP290 as well as other TZ proteins (TMEM67, Ahi1, and DZIP1/DZIPL1) disrupts ciliogenesis progression at the CV stage^59–66^. Based on these findings, it seems likely that a fully functional TZ is established as the CV develops into the ciliary sheath membrane. Structurally, the TZ has a Y-shape connecting the ciliary membrane to the microtubule doublets of the axoneme^67,68^. Thus, predicted transmembrane domain-containing TZ proteins such as TMEM67, TMEM216, TMEM231, and TMEM237 could link the ciliary membrane to the Y-shaped structures^3,69–73^. Moreover, given TMEM67 requirements at the CV stage, it, along with other TZ proteins, may be important for coordinating TZ and ciliary membrane assembly.

Establishing a functional TZ early in ciliogenesis could also be important for reorganizing the CV, from a spherical-like structure with a positive-curved cytoplasmic outward-facing membrane, into the ciliary sheath, with both positive and negative membrane curvature (Figure 1). The membrane opposed to the axoneme in the ciliary sheath and mature cilium also has negative curvature. Given the molecular gating function of the TZ, it could therefore help partition proteins and lipids in order to reorganize the membrane of the CV into the ciliary sheath. For example, the positive curvature–associated membrane-shaping factors PACSIN and EHD proteins would be predicted to interfere with establishing negative-membrane curvature at post-CV stages. Consistent with this idea, PACSIN1 and -2 and EHD1 and -3 are not detected in the mature cilium but are observed on the positively curved cytoplasmic surface of the ciliary pocket membrane^20,45^. Interestingly, the TZ protein FAM92 has an undefined BAR domain which could be important for establishing membrane curvature during ciliogenesis^74^. Further investigation of membrane-shaping mechanisms involved in CV-to-ciliary sheath transition is needed to better understand these processes. Clues to how membrane-shaping regulators affect primary cilium assembly may be found in studies of worm sensory cilia which can undergo dramatic membrane remodelling^75,76^.

## Coordinating growth of the ciliary membrane and the axoneme

The growth of the ciliary membrane and the axoneme microtubule doublets appears to be tightly coupled. Disruption of regulators of membrane trafficking and microtubule assembly blocks ciliogenesis at the CV stage or causes shortened cilia^5,20,77,78^. IFT proteins play a critical role in coordinating cilium axoneme growth via interactions with molecular motors and ciliary cargos^79,80^. IFT20 localizes to the Golgi^17^ and is recruited to the MC between the DAV and CV stages^20^ via the motor KIFC1^19^, suggesting that PCVs could be involved in the delivery of IFT20 to the MC. Interestingly, in *Chlamydomonas*, IFT-associated membrane vesicles were discovered near the base of the flagella and also contain axonemal components, suggesting that ciliogenic associations between ciliary membranes and axoneme occur outside of the cilium^81^. Finally, a more direct function of IFT on membrane trafficking was proposed for IFT172, which was shown to shape lipid membranes *in vitro*^82^.

Live cell imaging studies demonstrated that Rab8 accumulates in the growing cilium, supporting a role in the extension of the ciliary membrane^18,20^. Consistent with this idea, reduced PCV trafficking of Rabin8, regulated by the kinase NDR2, and ciliary accumulation of Rab8 are observed following the growth of the primary cilium^18,34^. Rab8- and IFT-coordinated functioning in cilium growth could be coupled by ELIPSA, which interacts with IFT20 and the membrane trafficking and ciliogenesis regulator Rabaptin5, which in turn interacts with Rab8^83^. Interestingly, IFT20 and Rab8 membrane trafficking regulation associations are further supported by studies in T cells, which despite lacking cilia require both proteins to regulate vesicular receptor recycling important for immune synapse function^84,85^.

Several other membrane trafficking regulators have also been linked to IFT and the extension of the cilium. Like Rab8, the exocyst regulator Sec10 accumulates in the cilium and associates with IFT proteins^86,87^. Another Ras superfamily member, Arl13b, functions in ciliogenesis and interacts with the IFT-B complex and mediates IFT-A retrograde transport on the axoneme^88^. Notably, Arl13b localizes to the ciliary membrane after the CV stage and has also been linked to exocyst ciliogenic function^89,90^, although the precise role of these proteins in cilium growth remains elusive. Rab35 was also recently reported to regulate cilium length by affecting Arl13b and lipid levels in the cilium^37^. Thus, Arl13b may serve as a bridge between IFT and membrane trafficking regulators in coordinating growth of the axoneme and the ciliary membrane. Rab23 has also been linked to IFT and ciliary growth^91^. Evidence for Rab23 requirements in ciliogenesis and cilium maintenance has also come from studies using Rab23 dominant-negative expression^39^ and investigation of its GTPase regulation by the GTPase-activating protein (GAP) Evi5L^24^ and the GEF complex Inturned (INTU) and Fuzzy, components of the CPLANE complex important for establishing planar cell polarity^91^. Interestingly, INTU and Fuzzy activate Rab23 for post-CV ciliogenic stages^91^ and INTU is known to modulate IFT ciliary trafficking^78,92^. Moreover, INTU interacts with another GTPase, RSG1, which is needed for assembly of the axoneme^78^. Together these observations point to multiple mechanisms whereby IFT couples axoneme and ciliary membrane growth in coordination with Rabs and other membrane trafficking regulators. These mechanisms are also likely important for IFT-dependent regulation of membrane cargo transport needed for ciliary signaling^79,80^.

Interestingly, very few reports have described the uncoupling of the growth of the primary cilium membrane and axoneme^66^. Ultrastructure analysis of fibroblasts from Joubert syndrome ciliopathy patients with CEP290 mutations displayed intracellular axonemes lacking ciliary membranes, yet DAVs appeared to be docked to the MC^66^. In contrast, depletion of the putative Rab-GAP Broad-minded (BROMI) showed detached and expanded ciliary membranes on one side of a seemingly normal axoneme in the zebrafish pronephros, although the target Rab has not been identified^93^.

## Fusion of the intracellular cilium with the cell surface

How the intracellular developing cilium emerges from the cell is another question that was recently investigated^45^. We discovered that the cilium can emerge from the cytoplasm via the formation of tubulovesicles organized from the positively curved surfaces of the CV and ciliary sheath membranes. These tubulovesicle structures are guided to the PM on microtubules, and upon fusion of these discrete membranes, an extracellular membrane channel (EMC) that exposes the ciliary membrane to the extracellular environment is formed. EHD and PACSIN proteins help reorganize these tubulovesicles^20,45^, and presumably Rabs, SNAREs, and molecular motors also function in this process as well. This study also showed Rab8 on these tubulovesicles, suggesting that factors needed for ciliogenesis could be delivered to the developing cilium from these structures, possibly originating from the PM. Thus, the intracellular and extracellular ciliogenesis pathways could use a similar mechanism for delivering ciliogenic cargo to the MC following docking to the PM.

## Conclusions

In this review, we examined new insights and models for membrane trafficking function in mediating discrete steps important for cilium assembly. Other membrane trafficking regulators have also recently been linked to ciliogenesis and ciliary trafficking, including the HOPS, ESCRT, and BLOC-1 complexes^94–96^. Thus, future studies examining these and other trafficking factors are important to address outstanding questions posed in this review, in particular relating to how PCVs specifically traffic and dock to the MC and how they organize to become the unique ciliary membrane in close coordination with the assembly of the ciliary TZ and axoneme. Although we did not focus on disease associations with membrane trafficking regulators, it is important to note that mutations in the membrane trafficking regulators are associated with ciliopathy: Arl13b (Joubert syndrome)^97^, Rab23 (Carpenter syndrome)^98^, and the exocyst subunits Exo84 (Joubert syndrome)^99^ and Sec8 (Meckel–Gruber syndrome)^100^. Thus, the investigation of fundamental ciliogenesis membrane trafficking process will undoubtedly enhance our understanding of cilia-related human disease.

## Abbreviations

CBY, Chibby; CV, ciliary vesicle; DA, distal appendage; DAP, distal appendage protein; DAV, distal appendage vesicle; ERC, endocytic recycling compartment; GAP, GTPase-activating protein; GEF, guanine nucleotide exchange factor; IFT, intraflagellar transport; INTU, inturned; KIFC1, kinesin-14; LPA, lysophosphatidic acid; MC, mother centriole; MPP9, M-phase phosphoprotein 9; PCV, preciliary vesicle; PM, plasma membrane; SDA, subdistal appendage; TTBK2, tau tubulin kinase 2; TZ, transition zone
